# A remarkable new species of the genus *Teinotarsina* (Lepidoptera, Sesiidae) from Okinawa-jima, Japan

**DOI:** 10.3897/zookeys.571.7780

**Published:** 2016-03-07

**Authors:** Sadahisa Yagi, Toshiya Hirowatari, Yutaka Arita

**Affiliations:** 1 Entomological Laboratory, Graduate School of Bioresource and Bioenvironmental Sciences, Kyushu University, Hakozaki 6-10-1, Fukuoka, 812-8581 Japan; 2 Entomological laboratory, Faculty of Agriculture, Kyushu University, 6-10-1 Hakozaki, Fukuoka, 812-8581 Japan; 3 Zoological laboratory, Faculty of Agriculture, Meijo University, Tempaku-ku, Nagoya, 468-8502 Japan

**Keywords:** Clearwing moth, mimicry, Sesiini, Taxonomy, Oriental region

## Abstract

A new species of long-legged clearwing moth *Teinotarsina aurantiaca* Yagi, Hirowatari & Arita, **sp. n.** is described from Okinawa-jima, the Ryukyus, Japan. The species is distinguishable at a glance from other related congeners by the remarkable orange scales ornamenting many parts of the body, such as antennae, palpi, legs, and wings. We hypothesize that the species acquired these differences as a result of convergence with toxic species (Batesian mimicry) or other mimics (Müllerian mimicry).

## Introduction

Clearwing moths, Sesiidae are diurnal and distributed all over the world. In recent years, many new species have been found in the eastern Palearctic, Oriental, Australian, Neotropical, and Ethiopian regions by attracting them with an artificial sex pheromone (Arita et al. 2009, Arita and Nasu 2011). In Japan, 44 species of Sesiidae have been recorded, and eight species are known from Okinawa-jima, the Ryukyus (Arita 2013). *Teinotarsina* Felder & Felder, 1874 is an Oriental tropical or subtropical genus, that comprises 11 species (five species with long hind legs) (Pühringer and Kallies 2016, Eda et al. 2015). This genus is characterized by the shortly ciliate antenna (Arita and Gorbunov 2002) and the valva of male genitalia being strongly arched dorsally, with a distinct falcate apex and relatively sparse simple setae (Eda et al. 2015). However, the biology of this genus is little known and few specimens have been collected because its members are not attracted by the artificial sex pheromones used so far. In May 2015, one of us (Yagi) collected an unusual clearwing moth, of which it was immediately obviously that this was a species not known in Japan, or Okinawa-jima, the Ryukyus. Examination of morphological characters including male genitalia revealed that it is a new species of *Teinotarsina* and distinguishable at a glance from other congeners by the characteristic feature of the beautiful ornamentation with orange scales.

In this paper, we describe this new species and illustrate the genitalia, comparing them with those of related species. We also briefly discuss why this species might have acquired such unique characters from the view point of mimicry.

## Material and methods

**Figure 1.**
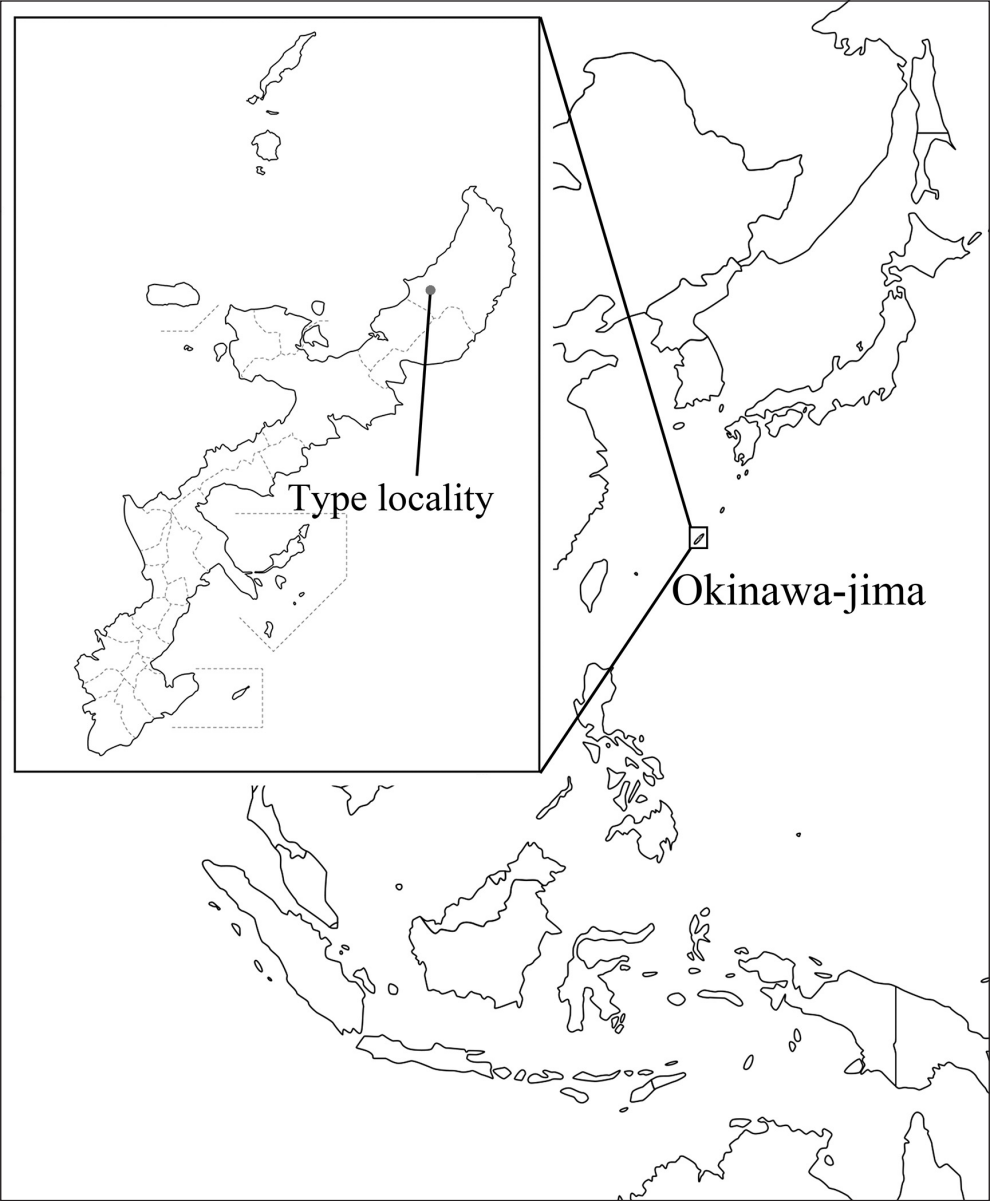
Map showing the type locality of *Teinotarsina aurantiaca* sp. n.

**Figure 2.**
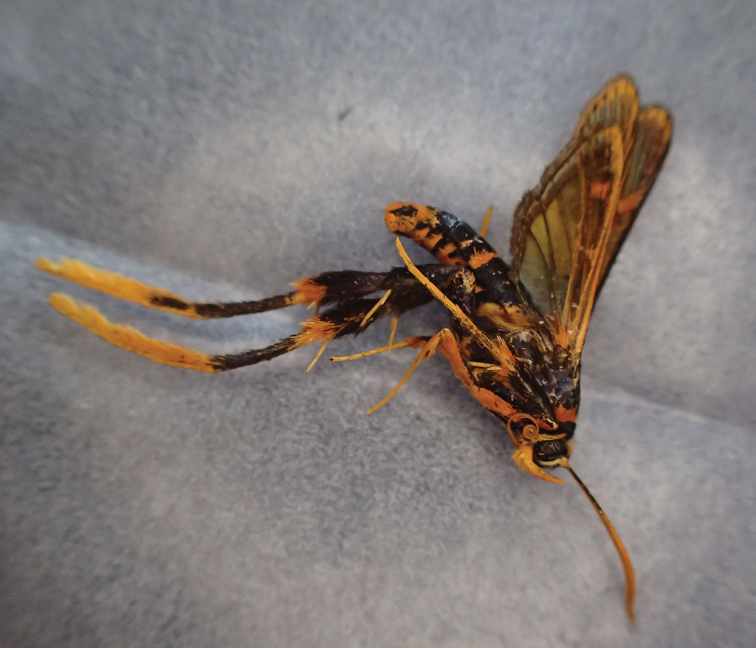
Male holotype of *Teinotarsina aurantiaca* sp. n., lateral view, before mounting.

Only one specimen of the new species was collected on 30 May 2015 in Okinawa-jima, the Ryukyus, Japan (26°43'46"N; 128°11'52"E) (Figs 1, 2). The specimen (holotype) is preserved in the Entomological Laboratory at the Kyushu University, Fukuoka, Japan. For preparation of the genitalia, the abdomen was detached and boiled in 10% aqueous KOH. Illustrations of the genitalia were prepared by using a Leica S8 APO microscope.

### Abbreviations

ELKU
 Entomological laboratory, Faculty of Agriculture, Kyushu University 


ETA
 External transparent area of forewing 


ATA
 Anterior transparent area of forewing 


PTA
 Posterior transparent area of forewing 


## Taxonomy

### Teinotarsina aurantiaca

Taxon classificationAnimaliaLepidopteraSesiidae

Yagi, Hirowatari & Arita sp. n.

http://zoobank.org/7532D2DB-E8B8-4961-BF9A-C317227932A0

Figs 3 , 4 , 5

#### Type material.

Holotype male (ELKU Type No.26), Hentona Kunigami-son, Kunigami-gun Okinawa Prefecture, Japan, 265 m, 30 May 2015, S. Yagi leg (ELKU).

#### Description.

**Figure 3.**
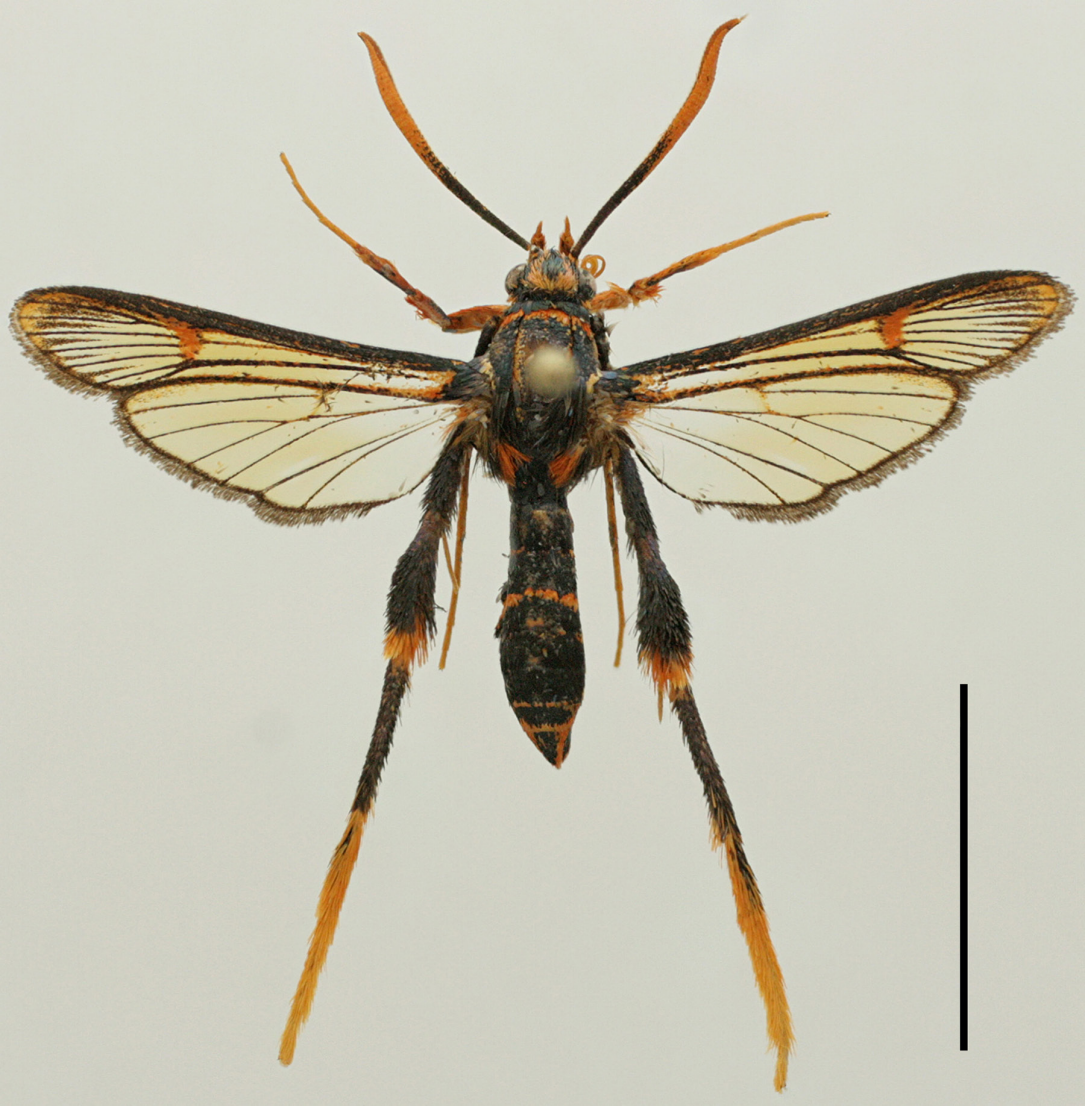
*Teinotarsina aurantiaca* sp. n. (Holotype). Scale bar: 10 mm.

Male (Figs 2, 3). Alar expanse 29.0 mm; forewing 12.3 mm; body length 14.2 mm; antenna 8.5 mm.

Head: frons black, with orange scales laterally; vertex black; pericephalic scales orange; collar with black hair-like scales, anteriorly with some orange hair-like scales; labial palps orange; scape orange; antenna dorsally covered with black scales in basal half, orange scales in apical half, ventrally naked, reddish brown, densely ciliate with yellow short sensory hairs; apical tuft yellow mixed with brown; proboscis yellowish orange.

Thorax: black; patagia black; tegula black, with orange scales anteriorly and long black hairs posteriorly; mesothorax black, with some orange scales laterally; metathorax black, with a tuft of orange hairs laterally.

Legs: fore coxa black with some orange scales basally; femur orange with black basally; fore tibia orange with black dorsally; fore tarsus yellowish orange; mid coxa, femur and tibia black with some orange scales distally; mid tarsus yellowish orange; hind coxa black with some orange scales distally; hind femur black; hind tibia in basal half with black fluffy hairs, in distal half basally black with dark violet-purple sheen, distally with black fluffy hairs and apical orange hairs; hind tarsus black in basal half, with yellowish orange hairs dorsally in distal half.

Abdomen: dorsally black with dark violet-purple sheen; tergite 5 with orange scales anteriorly, tergites 6-7 with narrow orange posterior margin; ventrally yellowish orange with dark violet-purple sheen. Sternite 3 with narrow orange anterior margin, sternite 4 with wide orange anterior margin, sternite 5 almost orange, sternites 6-7 orange with mixture of orange and black scales posteriorly; anal tuft short, orange mixed with black hair-like scales.

Forewing: basally transparent, other parts semitransparent with brownish sheen; costal and anal margins, CuA-stem black with dark violet-purple sheen, scattered with orange scales; discal spot yellow-orange; apical area narrow with yellowish orange scales; projections of dark brown scales from distal margin of forewing into cells of ETA; no projection into ATA and PTA; cilia dark brown with bronze sheen; dorsal margin mixed with black and orange scales.

Hindwing: basally transparent, other parts semitransparent with brownish sheen; veins and outer margin dark brown with orange scales; discal spot undeveloped; apical area with orange scales; outer margin narrow, about two-three times as narrow as cilia; cilia dark brown.

Male genitalia (Fig. 5). Tegumen well separated from uncus; uncus with a tuft of long setae posterodorsally, posteroventrally with a brush of long setae on each side, apical process relatively long, sharply-pointed ventrally; gnathos undeveloped; valva trapezoid with apical half broad; setae of inner surface short, thick, not pointed in median ventral area, long in apical half of central area, thin in dorsal area; sacculus developed; saccus rounded anteriorly; vinculum with a pair of projections posteriorly; Phallus posterodorsally broad; vesica with many small spine-like cornuti; manica with many minute spinules.

**Figure 4.**
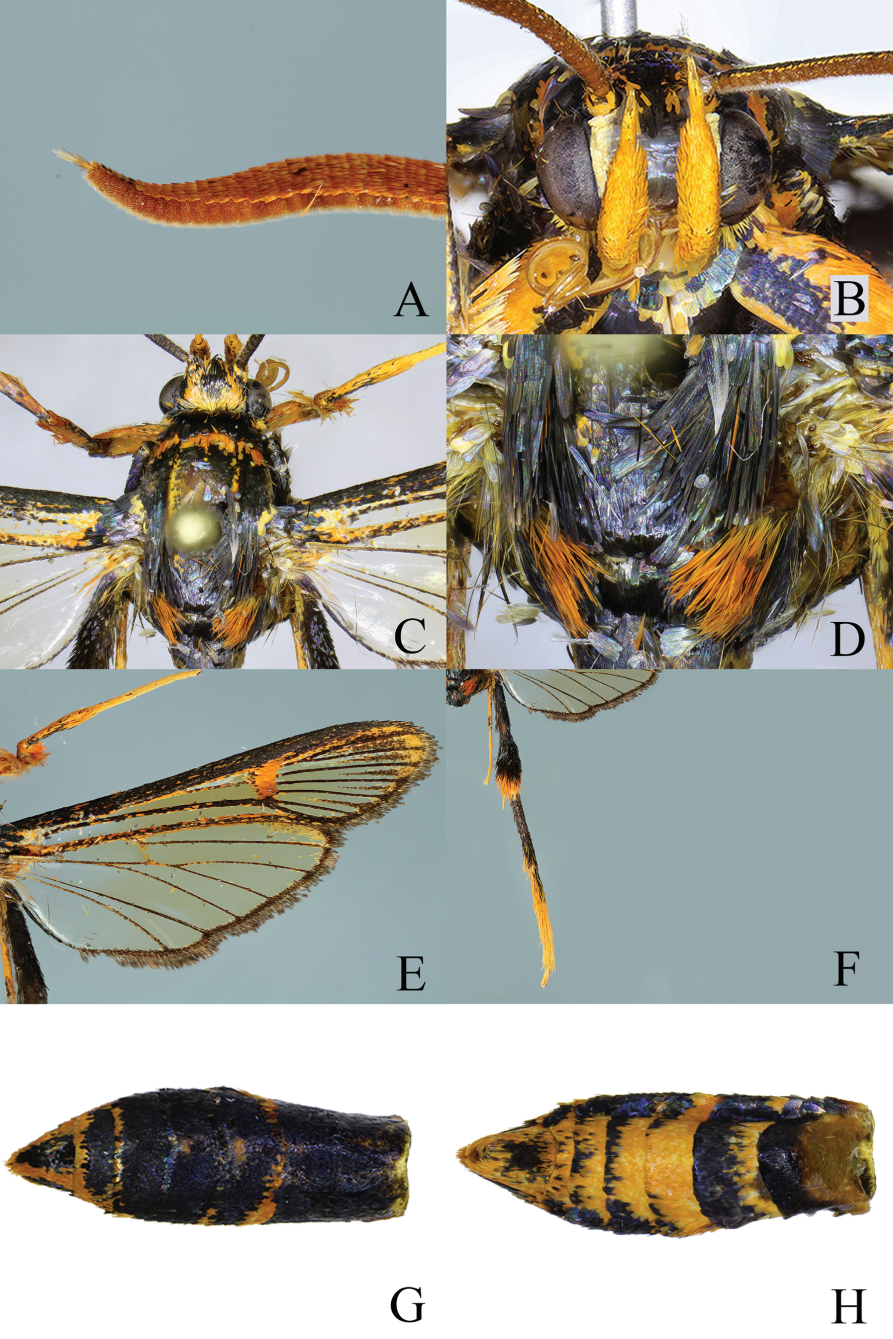
Body parts of *Teinotarsina aurantiaca* sp. n. (Holotype). **A** Apical part of antenna, lateral view **B** Head, frontal view **C** Thorax, dorsal view **D** Metathorax, dorsal view **E** Right wing **F** Hind leg, dorsal view **G** Abdomen, dorsal view **H** Ditto, ventral view.

**Figure 5.**
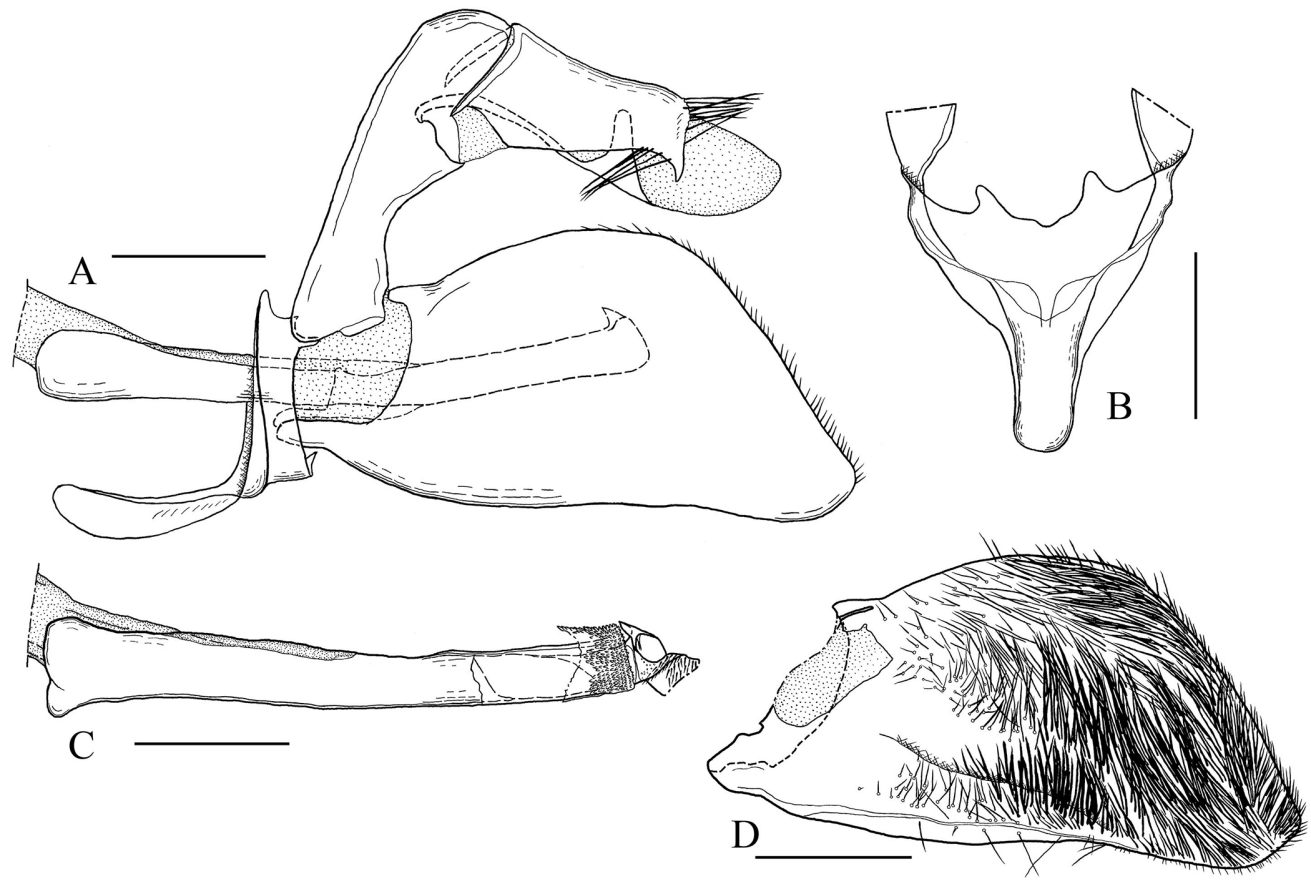
Male genitalia of *Teinotarsina aurantiaca* sp. n. (Holotype). **A** Whole genitalia, lateral view **B** Saccus and vinculum, ventral view **C** Phallus, lateral view **D** Valva, inner view. Scale bars: 0.5 mm.

Female. Unknown.

#### Etymology.

The species name “*aurantiaca*” an adjective, the female form of Latin aurantiacus (= orange), refers to the orange body of the new species.

#### Biology.

**Figure 6.**
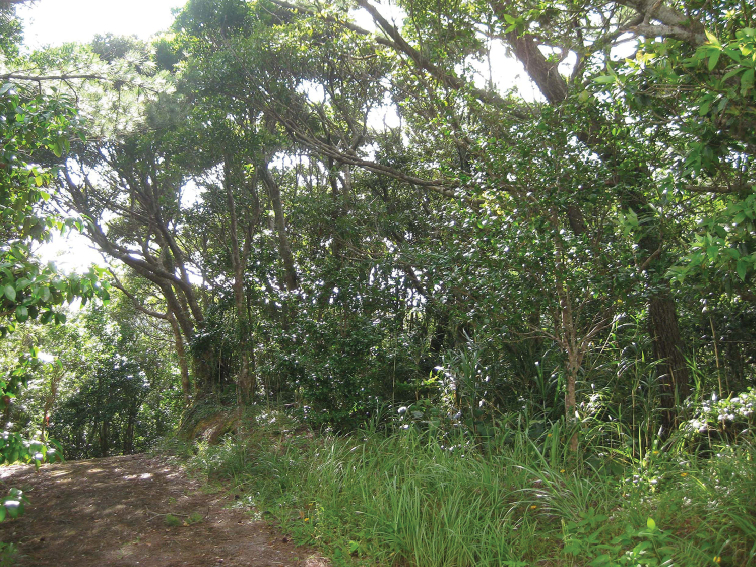
Habitat of *Teinotarsina aurantiaca* sp. n.

Unknown. The male holotype was collected on the roadside in a subtropical forest park “Shinrin Koen” (Fig. 6). In this habitat, *Castanopsis sieboldii* (Makino) Hatus. ex T.Yamaz. et Mashiba and *Pinus luchuensis* Mayr were dominant trees. The moth has been observed flying slowly at a height of approximately 0.7 m around 15:00.

#### Hostplant.

Unknown.

#### Distribution.

Okinawa-jima, Japan. Only known from the type locality.

#### Remarks.

The new species can be separated from *Teinotarsina longitarsa* Arita & Gorbunov, 2002, which occurs on Taiwan, by the orange coloration of the body, the relatively stout antennae with orange apical half (slender with a large white to pale yellow spot subapically in *Teinotarsina longitarsa*), and the fore- and hindwings which are semi-transparent with a brownish sheen (transparent overall in *Teinotarsina longitarsa*). The male genitalia of the new species are very similar to those of *Teinotarsina longitarsa* but are distinguishable from the latter by the following characters: 1) the setae on the ventral part of the uncus are longer than those in *Teinotarsina longitarsa*, 2) the apical process of the uncus is more sharply-pointed ventrally and relatively longer than that of *Teinotarsina longitarsa*, 3) the apical half of the valva is slightly broader than that in *Teinotarsina longitarsa*, 4) the anterior part of the aedeagus is broader than that of *Teinotarsina longitarsa*.

## Discussion

The family Sesiidae is generally considered to provide good examples of Batesian mimicry by mimicking hazardous insects such as bees and wasps (Duckworth and Eichlin 1974, Arita 2013)

The genus *Teinotarsina* often has antennae with a white spot subapically, as in *Teinotarsina longitarsa* and *Teinotarsina luteopoda* Kallies & Arita, 2004 and a blackish abdomen without a conspicuous stripe pattern. This suggests that Ichneumonidae (ichneumon wasps) might form a possible model for the genus *Teinotarsina*. However, in *Teinotarsina aurantiaca* sp. n., the coloration is distinctly different from the other species and this may indicate a different model species.

According to Terayama and Yamane (1999), in some wasp groups, the populations on Okinawa-jima are much darker (reddish or orange-tinged) in coloration than those on other islands of Nansei islands (chain of islands extending from southwestern Kyushu to Yonaguni-jima), to such a degree that they can be recognized as distinct subspecies. For instance, *Polistes rothneyi* Cameron, 1900 (Vespidae), which is generally maculated with bright yellow, is very dark on Okinawa-jima, so that most authors have mistaken it for the more melanistic *Polistes yakahamae* Radoszkowski, 1887. Many other hymenopterans, e.g. *Polistes japonicus* Saussure, 1858 and *Anterhynchium flavomarginatum* (Smith, 1852) (Eumenidae) show a comparable regional convergence. This syndrome can be seen not only in Hymenoptera but occurs also in other orders such as in Diptera: Syrphidae (Terayama and Yamane 1999).

In addition, on Okinawa-jima, species of the the sesiid moth genus *Nokona* Matsumura, 1931, including *Nokona rubra* Arita & Toševski, 1992 and *Nokona nigra* Arita, Kimura & Owada, 2009 are darker than other *Nokona* species on the main islands of Japan. Moreover, *Pennisetia insulicola* Arita, 1992 from the middle of the Ryukyus, is separated from *Pennisetia fixseni* (Leech, 1889) that occurs in Honshu, Kyushu, and Tsushima. Given this general trend, it is possible that Müllerian mimicry (with Aculeata as model) as well as Batesian mimicry (e.g. between Sesiidae and Syrphidae) play a role in Sesiidae on this island. This unique variety is perhaps derived from geographic isolation, e.g. by the channel between the Central and Southern Ryukyu areas (Muraji et al. 2012). Many species of Sesiidae on Okinawa-jima are superficially distinctly different from those in other areas. *Teinotarsina aurantiaca* sp. n. is also very different from other related species.

As for the other morphological characters, the male genitalia resemble closely those of *Teinotarsina longitarsa* from Taiwan, to which *Teinotarsina aurantiaca* is probably closely related. Further study is required to clarify the diversification of the genus *Teinotarsina*.

## Supplementary Material

XML Treatment for Teinotarsina aurantiaca
